# Receptor-Mediated Endocytosis and Brain Delivery of Therapeutic Biologics

**DOI:** 10.1155/2013/703545

**Published:** 2013-06-11

**Authors:** Guangqing Xiao, Liang-Shang Gan

**Affiliations:** Drug Metabolism and Pharmacokinetics, Biogen Idec, 14 Cambridge Center, Cambridge, MA 02142, USA

## Abstract

Transport of macromolecules across the blood-brain-barrier (BBB) requires both specific and nonspecific interactions between macromolecules and proteins/receptors expressed on the luminal and/or the abluminal surfaces of the brain capillary endothelial cells. Endocytosis and transcytosis play important roles in the distribution of macromolecules. Due to the tight junction of BBB, brain delivery of traditional therapeutic proteins with large molecular weight is generally not possible. There are multiple pathways through which macromolecules can be taken up into cells through both specific and nonspecific interactions with proteins/receptors on the cell surface. This review is focused on the current knowledge of receptor-mediated endocytosis/transcytosis and brain delivery using the Angiopep-2-conjugated system and the molecular Trojan horses. In addition, the role of neonatal Fc receptor (FcRn) in regulating the efflux of Immunoglobulin G (IgG) from brain to blood, and approaches to improve the pharmacokinetics of therapeutic biologics by generating Fc fusion proteins, and increasing the pH dependent binding affinity between Fc and FcRn, are discussed.

## 1. Introduction

This review is focused on the receptor-mediated endocytosis, transcytosis, and brain delivery of therapeutic biologics across the blood-brain-barrier (BBB). Transport of macromolecules across the BBB involves both specific and nonspecific interactions with proteins and receptors expressed on the luminal and/or the abluminal surfaces of the brain capillary endothelial cells. Endocytosis and transcytosis play important roles in the transport of macromolecules. The function of the neonatal Fc receptor (FcRn), the low density lipoprotein receptor related protein (LRP), the transferrin receptor (TfR), and the insulin receptor (IR) in regulating the endocytosis and transcytosis of immunoglobulin, peptides, and proteins across BBB has been studied. Due to the tight junction of BBB, brain delivery of traditional therapeutic proteins with large molecular weight is generally not possible. Over the past years, multiple methods have been attempted for brain delivery of drugs [1, 2]. Efficient brain delivery methods through receptor-mediated endocytosis and transcytosis have been developed based on the current knowledge of ligands and antibodies against the receptors on the brain endothelial cell surfaces. New peptides and antibodies with specific ability to cross the BBB have been reported. Angiopep-2, a peptide ligand of LRP1, was identified with high permeability across the BBB [3, 4]. Angiopep-2-conjugated systems have been developed by conjugating the therapeutic peptides and proteins to Angiopep-2 for efficient brain delivery [5, 6]. Two single domain antibodies (sdAb), FC5 and FC44, were also cloned using a phage-display library of llama single-domain antibodies [7, 8]. Owing to specific and high permeability across the BBB [9], FC5 and FC44 could be developed as the vectors for brain delivery. Molecular Trojan horse by fusing the therapeutic proteins to the monoclonal antibodies (MAb) against human insulin receptor (IR) or transferrin receptor (TfR) have been demonstrated to be the strategy for efficient brain delivery of therapeutic proteins [10, 11]. The brain delivery of a variety of therapeutic proteins has been evaluated with the molecular Trojan horses [11, 12].

The function and mechanism of FcRn in regulating immunoglobulin G (IgG) recycling have been well characterized. Because of the protective effects of FcRn against the lysosomal degradation of IgG, generating Fc fusion proteins and modulating the pH dependent affinity between Fc and FcRn has been approached to improve the PK of therapeutic antibodies [13, 14]. While in vitro and in vivo studies indicated the efflux of IgG from brain to blood is mediated by BBB FcRn [15, 16], conflicting results were also reported [17, 18]. These studies will be discussed in this review. 

## 2. Endocytosis and Transcytosis

Endocytosis is a process that cells engulf molecules. Endocytosis pathways can be divided into two categories, namely, phagocytosis and pinocytosis. Since these processes have been reviewed by Conner and Schmid [19] and Lin [20], the endocytosis pathways will be briefly described in this review. 

Phagocytosis is an endocytosis process called “cell-eating” which is involved in the acquisition of nutrients for some cells. It is a major mechanism to remove pathogens and cell debris in some immune systems. Phagocytosis is a specific form of endocytosis involving the vesicular internalization of solids which is distinct from other forms of endocytosis such as the vesicular internalization of various liquids. During the phagocytosis process, cells bind and internalize particulate substances with diameter larger than 0.75 *μ*m, such as small-sized dust particles, cell debris, microorganisms, and even apoptotic cells, and these processes involve the uptake of membrane areas larger than clathrin-mediated endocytosis and caveolae pathway. Phagosomes that are formed around the substances absorbed by phagocytosis migrate into the cytoplasma, mature through fusion with lysosomes, and subsequently form digestive vacuoles called phagolysosomes where substances are digested by the hydrolytic enzymes [21]. Phagocytosis is a process that occurs primarily in certain specialized cells such as macrophages, monocytes and neutrophils that are essential to remove large pathogens such as bacteria or yeast, or large debris. Because of that, phagocytosis is not expected to play an important role in the transcellular transport of therapeutic proteins. 

Unlike phagocytosis, pinocytosis is a fluid phase endocytosis process called “cell-drinking” or “fluid endocytosis”, in which cells form vesicle on the membrane and take small particles into the cell. The small particles are suspended within the small vesicles which subsequently fuse with lysosomes for digestion. After the macromolecules are taken up into the cells, a fraction of the endocytic vesicles may be expelled into external side, called exocytosis. Exocytosis can be on the same side as the endocytosis, or on the other side of the cells as the endocytosis which is termed as transcytosis. 

Pinocytosis is used primarily for the absorption of extracellular fluids. The size of the particles taken up by pinocytosis is smaller than that by phagocytosis. Unlike phagocytosis and receptor-mediated endocytosis, pinocytosis occurs in many kinds of cells, and is nonspecific in the substances it takes up; therefore it plays an important role in the transport of therapeutic proteins. Pinocytosis also works as phagocytosis, with the exception that phagocytosis is specific while pinocytosis is nonspecific in the substances they take up. Another difference between phagocytosis and pinocytosis is that in phagocytosis, cells engulf whole particles, break down by enzymes, and then absorb the broken-down products. During pinocytosis, in contrast, cells engulf already-dissolved or broken-down food. 

Pinocytosis can be further divided into three modes, namely, fluid-phase endocytosis, adsorptive endocytosis, and receptor-mediated endocytosis. There are also multiple pathways for pinocytosis, such as macropinocytosis, clathrin-mediated endocytosis, and caveolae-mediated endocytosis. Clathrin-mediated endocytosis is a process that the ligands bind into “clathrin coated pits” on the plasma membranes followed by small vesicle (approximately 100 nm in diameter) assembly. It occurs in virtually all cell types and takes up a variety of extracellular molecules, such as low density lipoprotein, transferrin, growth factors, and antibodies [19, 22, 23]. Caveolae are small flask-shape pits (approximately 50 nm in diameter) in the membrane that resemble the shape of a cave. They can constitute up to a third of the plasma membrane area of some cells, such as smooth muscle cells, fibroblasts, adipocytes, and endothelial cells [19, 24]. In contrast to clathrin-mediated endocytosis, the molecular mechanism of caveolae-mediated endocytosis still remains to be further elucidated. For example, it is not fully clear if and how the ligands taken up by caveolae-mediated endocytosis are digested [25]. 

In addition to clathrin- and caveolae-mediated endocytosis, clathrin- and caveolae-independent endocytosis exists. One example of clathrin- and caveolae-independent endocytosis is the internalization of human IgG in Caco-2 cells. In order to understand the mechanism of the absorption of therapeutic monoclonal antibodies by the human epithelial cells, the endocytosis and internalization of a human IgG into Caco-2 was examined. It is found the endocytosis of the human IgG into Caco-2 cells was pH, temperature, and ATP dependent. In addition, caveolin-dependent endocytosis inhibitors Nystatin and Indomethacin had no significant effects on the cell association and binding of human IgG to the Caco-2 cells, indicating that the internalization is a clathrin- and caveolin-independent endocytosis [26].

Fluid-phase endocytosis is a nonspecific process driven by the concentration of the extracellular side. It does not require ligand binding to cell surface membrane, therefore it is a non-competitive process, and it is not an efficient way of endocytosis [19]. Uptake of fluid by cells can occur either by micropinocytosis within vesicles (<0.1 *μ*m in diameter) or by macropinocytosis within vacuoles (approximately 0.5–5.0 *μ*m in diameter). The macrophage of the native low-density lipoprotein (LDL) is a fluid-phase pinocytosis. The endocytosis is receptor independent, and the uptake is inhibited by macropinocytosis inhibitors, such as phosphatidylinositol 3-kinase inhibitor and LY294002, but not by micropinocytosis inhibitors such as Nystatin and Filipin. The taken up of LDL in the fluid phase macropinocytosis without receptor-mediated binding is a novel endocytosis pathway that generates macrophage foam cells [27, 28]. The endocytosis of IgG into Caco-2 cells is also a process of macropinocytosis since macropinocytosis inhibitors such as cytochalasin B and 5-(N-ethyl-N-isopropyl) amiloride significantly decreased the uptake of the human IgG at pH 6.0 [26].

Adsorptive endocytosis requires a ligand cell surface interaction and is triggered by an electrostatic interaction between the positively charged micromolecules or proteins and negatively charged plasma membrane surface. Micromolecules or proteins interact with the cell surface membrane and are concentrated before being internalized. Adsorptive endocytosis is a nonspecific process and is often via the clathrin-mediated mode [19, 28]. The cell uptake of the iron oxide nanoparticles into the Caco-2 cells is an adsorptive endocytosis process [29]. Adsorptive endocytosis based brain delivery of cationic proteins and cell penetrating peptides (CPPs) has been attempted. The method is based on the potential of the brain capillary endothelial cells to bind and uptake cationic molecules at the luminal surface and subsequently exocytosis the molecules to the abluminal surface. Two main families of cationic CPPs belonging to the Tat-derived peptides and Syn-B vectors have been extensively used in the delivery of a large variety of small molecules as well as proteins across cell membranes in vitro and across the BBB in vivo. However the usage of CPPs is associated with issues such as toxicity and immunogenicity due to the cationization strategy, and the instability of the peptide vectors in biological media [30].

Receptor-mediated endocytosis is a specific process for cells to take up small and large molecular ligands, including hormones, growth factors, enzymes, and plasma proteins. Due to the limited number of the receptors on the cell surface, receptor-mediated endocytosis is normally a saturable process. One example of saturation for receptor-mediated endocytosis/clearance is the nonlinear pharmacokinetics (PK) of the recombinant human erythropoietin (rh-EPO). Study in rats showed the total body clearance of the rh-EPO decreased as the dose increased from 0.2 to 5 *μ*g/kg following a single intravenous administration. Clear saturation was observed on the uptake clearance of ^125^I-rh-EPO by the target tissues, such as bone marrow and spleen. The tissue uptake clearance of ^125^I-rh-EPO by bone marrow and spleen was reduced due to the competition with a large dose (1 *μ*g/kg) of unlabeled rh-EPO given by subcutaneous administration [31]. Another example is the clearance of 2F8, a therapeutic monoclonal antibody (MAb) against the epidermal growth factor receptor (EGFR). Rapid receptor-mediated internalization of 2F8 by EGFR-overexpressing cells was observed from in vitro studies. In vivo study in cynomolgus monkeys showed the accelerated clearance of 2F8 occurred at low dose but not at high dose, which could be explained by the saturation of EGFR receptor-mediate 2F8 endocytosis. It is noteworthy that the saturation of EGFR mediated endocytosis in normal tissues did not predict the saturation in tumor tissue as the local antibody concentrations in EGFR-overexpressing tumors may be more rapidly reduced by antibody internalization [32]. For glycoproteins, significant receptor-mediated clearance may occur via interactions with sugar-specific receptors, such as asialoglycoprotein receptor or mannose receptor [33, 34]. *Streptococcus pneumonia*, which has a capsule rich in mannosyl residues, is the most common cause of rhinosinusitis that may evolve to meningitis. In vitro studies indicated the endocytosis of *Streptococcus pneumonia* to olfactory ensheathing cells is mediated via the mannose receptor [34]. A member of Ca2^+^ dependent lectin family is the mannose receptor which is mainly expressed on the surface membranes of macrophages and hepatic endothelial cells. They can mediate the uptake of glycoproteins that contain terminal mannose, N-acetylglucosamine, and fucose residues [35]. Tissue plasminogen activator (TPA), a protein involved in the breakdown of blood clots, has been used clinically to treat embolic or thrombotic stroke. However, the clinical application of TPA is complicated by its fast clearance from the bloodstream to liver due to mannose receptor expression on the endothelial liver cells and the LDL receptor-related protein (LRP) expression on parenchymal liver cells. To address whether the TPA clearance can be reduced by inhibiting the receptor-mediated endocytosis of TPA, a series of cluster mannosides was synthesized. A cluster mannoside carrying six mannose groups (M6L5) displayed high affinity to the mannose receptor. Pre-injection of M6L5 (1.2 mg/kg) reduced the clearance of ^125^I-TPA in rats by 60% resulting from specific inhibition of mannose receptor-mediated endocytosis into endothelial cells. Blockade of LRP by a 39-kD receptor-associated protein (GST-RAP) also inhibited TPA clearance by 60%. Pre-injection of both M6L5 and GST-RAP almost completely blocked the liver uptake of TPA and reduced the clearance by about 10 times. The study suggested that prolonged therapeutic effect of TPA can be maintained by coadministration of the M615 and GST-RAP [36].

Receptor-mediated endocytosis has also been utilized for efficient drug delivery to the target cells with high expression of the receptors. For examples, transferrin receptor (TfR) and insulin receptor (IR) mediated endocytosis systems have been used for small molecules and therapeutic protein delivery [1, 11, 12, 37]. TfR is expressed at a higher level in bronchial epithelial cells compared to their alveolar counterparts, and the expression of TfR in cancerous origin is higher than the healthy alveolar epithelial cells in particular. Transferrin-conjugated liposomes is, therefore, a good candidate as drug delivery systems for inhalation therapy of lung cancer [38]. The delivery of adriamycin to resistant human tumor cells is also mediated by TfR mediated endocytosis. In this case, adriamycin was covalently conjugated to transferrin. This conjugate, Trf-adr, was found to bind to TfR receptor in a variety of human tumor cell lines and exhibited more potency against resistant human tumor cell lines than sensitive cell lines. In vivo study in advanced tumor bearing nude mice indicated that the Trf-adr conjugate showed prolonged exposures than the unconjugated adriamycin [39]. 

Unlike therapeutic monoclonal proteins that target the cell surface receptors, the PK of therapeutic proteins that target the soluble proteins in the blood appears to be linear. For example, the PK of adalimumab, a fully human anti-tumor necrosis factor-*α* (anti-TNF*α*) monoclonal antibody, is linear over a wide dose range [40]. Following single intravenous injections of ascending doses from 0.5 to 10 mg/kg, adalimumab systemic drug exposure increased linearly with the increase in dose. The total serum clearance, the volume of distribution, and the terminal half-life were similar within the dose range.

## 3. The Low Density Lipoprotein Receptor Related Protein Mediated Brain Delivery via Angiopep-2

The low density lipoprotein receptor related protein (LRP) has been reported to mediate the endocytosis of A*β* amyloid peptides across the BBB [41–43]. Aprotinin, a basic pancreatic trypsin inhibitor, which contains the Kunitz protease inhibitor (KPI) sequence, is a ligand of LRP [44, 45]. In vitro and in vivo studies indicated that the transport of Aprotinin across the BBB is mediated by LRP [46]. By aligning the amino acid sequence of Aprotinin with the Kunitz domain of human proteins, a family of peptides, named Angiopeps, were identified [4]. Endocytosis study using the bovine brain capillary endothelial cell (BBCEC) monolayer, an in vitro BBB model, showed these peptides have good ability to transport across the monolayers. Among the peptides, Angiopep-2 showed the best ability, with 3–7 times higher endocytosis in comparison to Aprotinin [3, 4]. In situ brain perfusion also showed the brain distribution of Angiopep-2 is much higher than that of Aprotinin. 

LRP1 is a receptor with multiple functions and is expressed ubiquitously. Western blot analysis indicated only LRP1, but not LRP2, is expressed in human endothelial cells [4]. In vitro studies showed the apical-to-basolateral transport of Angiopep-2 across the BBCEC monolayers was inhibited by the receptor associated protein (RAP), a ligand of LRP1 [4]. In addition, the LRP1 mediated uptake of RAP was inhibited by both Angiopep-2 and Aprotinin in a concentration dependent manner [3]. Additional studies showed that Angiopep-2 had a high level of accumulation in parenchymal. The transport was not inhibited by the Pgp inhibitor CsA, but by alpha(2)-macroglobulin, a specific ligand for LRP1. Fluorescent microscopy also revealed that Alexa488-Angiopep-2 colocalized with LRP1 in the brain endothelial cell monolayers [3]. Overall, these results suggest that Angiopep-2 transport across the BBB is mediated by LRP1.

High BBB permeability ability associated with Angiopep-2 enables it to be utilized as a vehicle for BBB delivery of small molecules, DNAs, and proteins. A dual-drug delivery system to brain tumor was developed based on PEGylated oxidized multi-walled carbon nanotubes (O-MWNTs) modified with Angiopep-2 (O-MWNTs-PEG-ANG) [47]. Following the LRP1 mediated Angiopep-2 endocytosis across the BBB, the drug binds and accumulates in the tumor cells. The system has been used to delivery doxorubicin across the BBB. Study with mice indicated that DOX-loaded O-MWNTs-PEG-ANG (DOX-O-MWNTs-PEG-ANG) showed higher anti-glioma effects, better biocompatibility, and lower cardiac toxicity than those of the unmodified DOX [47]. GRN1005 is another Angiopep-2-paclitaxel conjugated drug that targets the low-density lipoprotein receptor-related protein 1. Clinical studies were conducted to evaluate the safety, tolerability, PK, and efficacy in patients with advanced solid tumors. GRN1005 has been shown to be well tolerated and showed activity in heavily pretreated patients with advanced solid tumors [48].

The PAMAM-PEG-Angiopep/DNA nanoparticles system, constructed by conjugating polyamidoamine (PAMAM) to polyethyleneglycol (PEG) and the DNA, has been developed to specifically deliver DNA to brain glioma for gene therapy. Both in vitro and in vivo results indicated the accumulation of PAMAM-PEG-Angiopep/DNA nanoparticles in the brain, especially the tumor site, was higher than that of PAMAM-PEG/DNA and PAMAM/DNA nanoparticles. PAMAM-PEG-Angiopep/DNA NPs can be a potential non-viral delivery system for gene therapy of glial tumor [49]. An Angiopep-conjugated poly(ethylene glycol)-co-poly(epsilon-caprolactone) nanoparticles (ANG-PEG-NP, also termed as PEG-PCL-NP) system has also been developed to specifically deliver drugs to brain [5, 6, 50, 51]. By fusing the EGFP-EGF1 protein to the cascade, the system precisely delivered EGFP-EGF1 to the brain neuroglial cells. In vitro studies demonstrated that both the bEnd.3 cells and the neuroglial cells had a higher uptake of Angiopep-2 and EGFP-EGF1 conjugated nanoparticles (AENP) as compared to the unmodified nanoparticles. Ex vivo imaging showed that AENP had higher accumulation in the brain over the unmodified nanoparticles or EGFP-EGF1-nanoparticles [6].

The mechanism of ANG-PEG-NP delivery across the BBB has been investigated in mice by labeling the ANG-PEG-NP with a fluorescence probe Rhodamine B isothiocyanate (RBITC). The study showed that after injection in mouse caudal vein, ANG-PEG-NP was delivered to mouse brain, with a higher accumulation in the cortical layer, lateral ventricle, third ventricles, and hippocampus than that of PEG-NP. The delivery was a caveolae- and clathrin-mediated endocytosis process, and the process was time, concentration, and energy dependent. The accumulation was inhibited by LRP ligands such as Angiopep-2 and aprotinin, confirming endocytosis was mediated by LPR1 receptor [51].

## 4. Transferrin and Insulin Receptor-Mediated Brain Delivery with Molecular Trojan Horses

**Figure 1:**
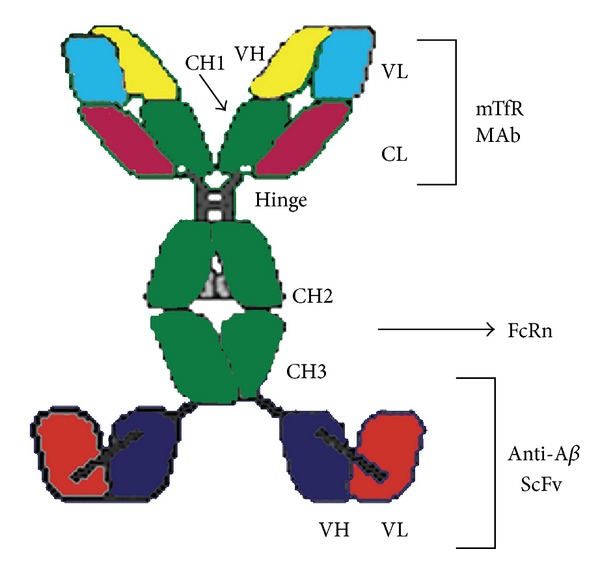
The cTfRMAb-ScFv fusion protein is formed by fusion of the variable region of the heavy chain (VH) of the rat 8D3 MAb against the mouse transferrin receptor (mTfR) (yellow) to the amino terminus of mouse IgG1 constant (C) region (green), and fusion of a single chain Fv (ScFv) antibody against the A amyloid peptide to the carboxyl terminus of the heavy chain C-region. The light chain is composed of the variable region of the light chain (VL) of the rat 8D3 MAb (light blue) and the mouse kappa light chain C-region (CL) (dark red). The heavy chain constant region is composed of 4 domains: CH1, hinge, CH2, and CH3. The CH2-CH3 interface is the binding site for the neonatal Fc receptor (FcRn). The ScFv is composed of the VH (dark blue) and the VL (light red) derived from the anti-AMAb (adapted from Figure 1, [52]).

Due to the tight junction of BBB, brain delivery of traditional therapeutic proteins with large molecular weight is generally not possible. There are multiple pathways that macromolecules can be taken up into cells through both specific and nonspecific interactions with proteins and receptors on the cell surface. Among the ways to enhance brain delivery, molecular Trojan horse (MTH) method has demonstrated as a strategy to efficiently delivery therapeutic proteins to brain through receptor-mediated endocytosis and transcytosis. Of the receptors expressed in the brain endothelial cells, insulin receptor (IR) and transferrin receptor (TfR) are the mostly used with the molecular Trojan horses. The molecular Trojan horse is generally constructed by fusing a therapeutic protein to each of the heavy chain of a genetically engineered chimeric monoclonal antibody against the TfR or IR. A representative structure of fusion protein through Trojan horse strategy is shown in Figure 1 [52]. The brain delivery of a variety of therapeutic proteins has been evaluated via the Trojan horse strategy [11, 12].

Both IR and TfR are expressed on the brain capillary endothelial cells [1, 11, 12, 53]. The expression of TfR on both the luminal and abluminal sides of the endothelial cells has also been demonstrated using freshly isolated rat brain capillaries [53]. 

The molecular Trojan horse method was based on the fact that receptors expressed on the BBB can mediate the endocytosis and transcytosis of monoclonal antibodies against the receptors. The ability of MAb83-14, a monoclonal antibody against human IR, to undergo transcytosis was demonstrated in rhesus monkeys. Following a single intravenous injection to rhesus monkeys, 3.8% of dosed MAB83-14 was delivered to brain whereas no brain uptake was observed of the control monoclonal antibody [54]. In another study, the rat brain distribution of OX26, a murine monoclonal antibody against rat TfR, was 18 times greater than the distribution of the control mouse immunoglobulin G2a [55]. Similarly, TfR mediated OX26 transcytosis from blood to brain was demonstrated in rats [56]. Collectively, these studies supported the application of TfR and IR based molecular Trojan horses for brain delivery.

For IR and TfR, it is thought that after ligand binding, the receptor-ligand complex undergoes endocytosis at the luminal membrane followed by the migration of vesicle across the cytoplasma and ends by the fusion of the vesicle to the abluminal side of the endothelial cells. The ligand is subsequently released from the receptor; that is, the ligand is transported from the luminal membrane to the abluminal membrane. Furthermore, the study of the TfR mediated efflux of both apotransferrin and holo-transferrin across BBB in rats provided evidence that TfR can mediated transcytosis across BBB in both blood-to-brain and brain-to-blood directions [57].

The TfR and IR receptor-mediated endocytosis are generally species specific. To compare the brain delivery of 8D3 and RI7-217, two murine monoclonal antibodies against the mouse TfR, with OX26, the murine monoclonal antibody against the rat TfR, a study was conducted in mice. Both 8D3 and RI7-217 antibodies showed high transport across the mouse BBB, with brain uptake of 3.1% and 1.6% of the injected dose [(ID)/g], respectively. In contrast, the mouse brain uptake of the OX26 antibody was 25–50 times lower, with only 0.06% ID/g of the injected dose [58]. These studies highlighted the selection of right antibodies for the molecular Trojan horse based brain delivery. The application of the molecular Trojan horse method to deliver therapeutic proteins is primarily led by Pardridge and his colleagues. A comprehensive list the therapeutic proteins that are delivered to brain with the molecular Trojan horse method can been found in the review article [12]. Some of the therapeutic proteins are discussed here. 

### 4.1. Tumor Necrosis Factor Receptor

Tumor necrosis factor *α* (TNF*α*) is a proinflammatory cytokine that is synthesized in brain within 1 hour of an acute experimental ischemic stroke. The leading decoy receptor-type TNF inhibitor (TNFI) is etanercept, which is widely used to suppress TNF*α* action in inflammation in peripheral organs [59]. However etanercept cannot be developed for the treatment of brain stroke since it cannot penetrate the BBB. To enable the delivery of the biologic TNFI, the type II human TNF receptor (TNFR) was fused to the genetically engineered chimeric monoclonal antibody (MAb) against the mouse TfR, designated as cTfRMAb-TNFR fusion protein [60]. Forty-five minutes after intravenous administration at 1 mg/kg, the fusion protein caused 40–50% reduction in hemispheric, cortical, subcortical stroke volumes, and neural deficit. As a control, treatment of 1 mg/kg etanercept had no significant changes in either stroke volume or neural deficit score. 

**Figure 2:**
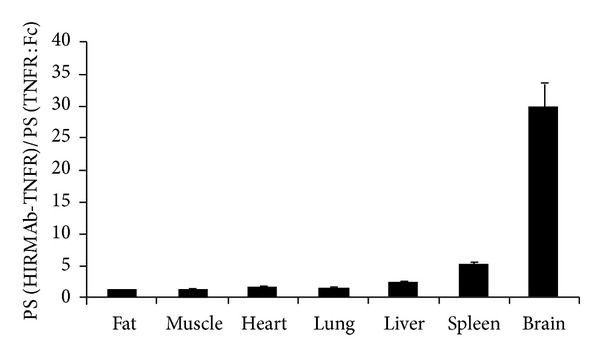
Ratio of the organ PS product for the HIRMAb-TNFR fusion protein, over the organ PS product for the TNFR:Fc fusion protein, is plotted for each organ. The ratio for brain is the mean of the values for frontal gray matter, frontal white matter, cerebellar gray matter, and cerebellar white matter, which varied between 22 and 37 (adapted and modified from Figure 8, [61]).

TNF*α* also plays a role in the pathology of brain disorders, including Parkinson's disease, Alzheimer's disease, and depression. Deletion of TNFR in mice produced resistance to Parkinson's disease induction neurotoxins [62]. Leading TNF*α* inhibitors included a TNF decoy receptor Fc fusion protein (infliximab), a chimeric anti-TNF*α* MAb. A fusion protein between TNFR and human IR, HIRMAb-TNFR, has been engineered [61]. The brain uptake of the fusion protein was much higher than that of TNFR-Fc. The permeability-surface area (PS) product of HIRMAb-TNFR to TNFR-Fc was about 30 for brain, but much lower from other peripheral organs (Figure 2). The HIRMAb-TNFR fusion protein maintained both the high affinity to HIR to mediate brain delivery, and the affinity to human TNF*α* to suppress the cytotoxic effects of this cytokine. While the TNF decoy receptor Fc fusion protein (infliximab) showed prolonged residence time in blood, it did not cross the BBB, probably due to the BBB FcRn mediated efflux from brain to blood [16].

### 4.2. Anti-A*β* Amyloid Peptide Antibodies

The fusion of a single chain Fv (ScFv) antibody against A*β* amyloid peptide and the rat 8D3, a MAb against the mouse TfR, was engineered by fusing the ScFv antibody to the carboxyl terminus of the heavy chain of the mouse/rat chimeric monoclonal antibody against TfR [52]. The fusion antibody, cTfRMAb-ScFv, has three function groups: binding to TfR for brain delivery, binding to the amyloid plaque target, and binding to FcRn to maintain prolonged half-life and to remove the amyloid plaque from brain to blood. The study in mice indicated the fusion protein not only enabled the rapid uptake of ScFv to access the amyloid plaque in the brain, but also rapid removal of the plaque from the brain. The function of the fusion protein to remove A*β* amyloid peptides from brain was also demonstrated in another mice study, where the treated mice showed 40% reduction in the brain A*β*-42 level without any elevated A*β* amyloid peptide concentration in plasma [63]. The function of the fusion protein to remove the A*β* amyloid peptide from brain to blood could be due to TfR mediated transcytosis from brain to blood direction since it has been reported that TfR can mediated the transcytosis of circulating transferrin in both blood-to-brain and brain-to-blood directions [57].

### 4.3. Anti-Aspartyl Protease *β*-Site APP Cleavage Enzyme 1

Aspartyl Protease *β*-site APP Cleavage Enzyme 1 (BACE1) is a prime therapeutic target for Alzheimer's disease. The therapeutic effect of an anti-BACE1 antibody in inhibiting A*β* production has been demonstrated in vivo [64]. To enhance the brain delivery of the anti-BACE1 antibody, a bi-specific antibody was generated by fusing a low affinity anti-TfR antibody to a high affinity anti-BACE1 antibody [65]. The selection of an anti-TfR antibody with low affinity but not high affinity was based the PK results in mice which indicated that, compared to the anti-TfR antibodies with higher affinity, anti-TfR antibodies with lower affinity showed increased brain uptake and broader distribution in brain parenchyma, likely due to the faster dissociation from the TfR because of the lower affinity, therefore higher transcytosis across the BBB.

### 4.4. Glial Cell Line Derived Neurotrophic Factor

Glial cell line derived neurotrophic factor (GDNF) is part of the transforming growth factor *β* (TGF*β*) superfamily and has a role in the development and maintenance of mesencephalic dopaminergic neurons. It has showed neuroprotective and restorative properties in Parkinson's disease animal models [66, 67]. However being a large molecule, GDNF cannot penetrate the BBB and has to be administrated by intra-cerebral injection. GDNF was fused to the heavy chain of a chimeric monoclonal antibody against mouse TfR, named cTfRMAb-GDNF. The fusion protein showed remarkable neuroprotective effects in the experimental Parkinson's disease mice which were induced by the intra-striatal injection of 6-hydroxydopamine. Following daily intravenous injection of the fusion protein for 3 weeks, the treated mice showed a 44% decrease in apomorphine-induced rotation, a 45% reduction in amphetamine-induced rotation, a 121% increase in the vibrissae-elicited forelimb placing test, and a 272% increase in striatal tyrosine hydroxylase enzyme activity at 3 weeks after toxin injection [68].

### 4.5. Erythropoietin

Erythropoietin (EPO) is a neurotrophic factor that could be developed as a drug for brain disorders. HIRMAb-EPO was engineered by fusing human EPO to the carboxyl terminus of the heavy chain of a chimeric monoclonal antibody against the human IR [70]. The fusion protein and HIRMAb bind HIR with equal affinity. Study on rhesus monkeys showed that while the unmodified EPO did not cross BBB, the fusion protein was selectively delivered to the brain compared to the peripheral organs. The PS product ratio between HIRMAb-EPO and the unmodified EPO increased significantly (approximately 3–10 times) in brain tissues than other organs, such as spleen, liver, heart, and kidney. 

### 4.6. Therapeutic Proteins for Mucopolysaccharidosis

 Mucopolysaccharidoses (MPS) are a group of metabolic disorders caused by the absence or malfunction of lysosomal enzymes. MPS affects CNS; however enzyme replacement therapy is not effective for the brain disease since the therapeutic proteins, such as iduronate-2-sulfatase (IDS) for MPS type II, do not cross the BBB and cannot be delivered to the brain [71, 72]. The fusion protein of IDS with HIRMAb was engineered [73]. The fusion protein is a bi-functional molecule, retained both the binding affinity to IR and the high IDS enzyme activity. The HIRMAb-IDS fusion protein was efficiently taken up by the MPS type II fibroblasts which resulted in 84% reduction of glycosaminoglycan accumulation. 

However not all fusion proteins maintained the function to both the receptor and the target. *B*-Glucuronidase (GUSB) is a lysosomal enzyme that could be developed as a therapeutic protein for either antibody directed enzyme pro-drug therapy or enzyme replacement therapy of MPS type VII. Being unable to cross BBB, human GUSB was reengineered as a fusion protein, either to the carboxyl terminal or the amino terminal of the heavy chain of the monoclonal antibody against human IR, named as HIRMAb-GUSB and GUSB-HIRMAb, respectively [74]. The HIRMAb-GUSB fusion protein maintained the HIR binding activity but lost the GUSB enzyme activity. On the other hand, the GUSB-HIRMAb maintained the GUSB enzyme activity but lost the HIR binding activity. 

Brain delivery through receptor-mediated endocytosis is associated with the administration of receptor ligands, which could interfere the intended function of the receptors. The effect of chronic high dose administration of HIR fusion protein was evaluated in cynomolgus monkeys [10]. In this study, lysosome enzyme iduronidase (IDUA), a gene therapy drug, was fused to the carboxyl terminal of a monoclonal antibody against human IR (HIRMAb-IDUA). The effect of weekly dose of HIRMAb-IDUA at 3, 9, and 30 mg/kg for 6 month on the plasma glucose and long term glycemic control was evaluated. The study showed that while the fusion protein in general did not affect the glucose clearance from plasma, the glucose distribution in CSF and plasma, and the glucose tolerance, chronic dose at 30 mg/kg of the fusion protein had weak insulin agonist properties and caused hypoglycemia.

## 5. Transport of FC5 and FC44 across BBB

Single domain antibodies (sdAb) are the humoral immune response for camels, dromedaries, and llamas. Unlike whole antibodies, sdAbs are formed by two heavy chains but no light chains. The molecular weight of sdAbs is only 12–15 kDa, much smaller than the Fab fragment of the whole antibody, or the single-chain variable fragment (ScFv). However similar to the whole antibodies, sdAbs are able to bind selectively to a specific antigen [75]. Two sdAbs, FC5 and FC44, were selected, sequenced, and subcloned using a phage-display library of llama single-domain antibodies [7, 8].

**Figure 3:**
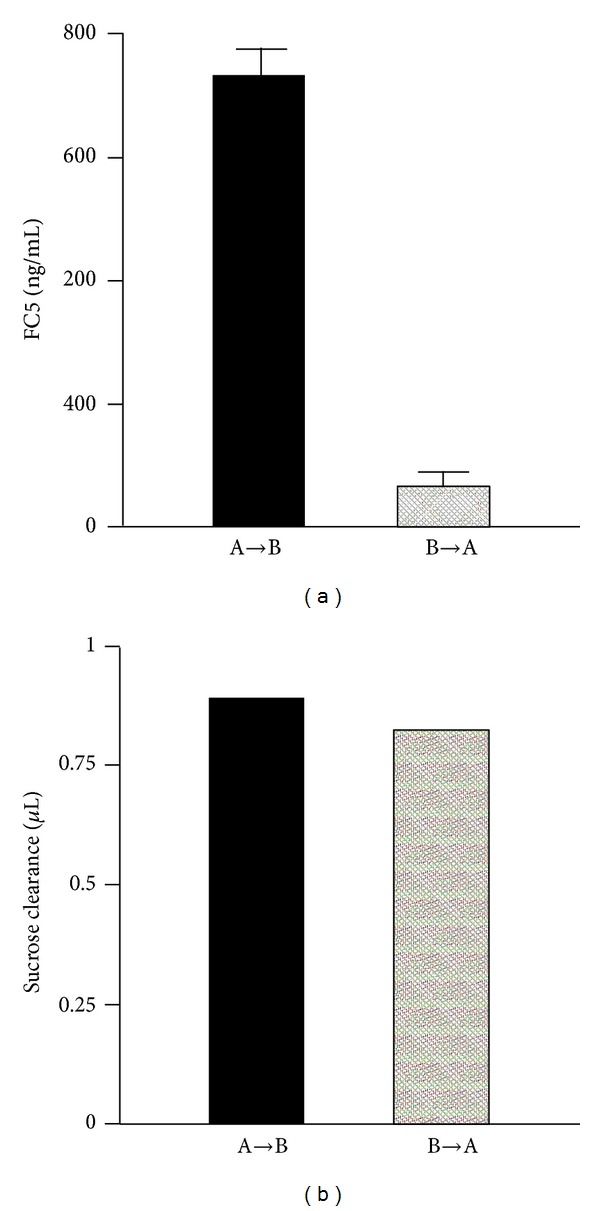
(a) Polarized transmigration of FC5 across HCEC monolayers. Transport studies were initiated by adding 10 *μ*g/mL FC5 to either apical (A to B) or basolateral (B to A) compartment and the amount of FC5 in the opposite compartment was determined after 30 min. (b) [14C] Sucrose distribution across the same HCEC monolayers was used as internal control for paracellular transport (adapted and modified from Figure 1, [69]).

The ability of FC5 and FC44 to transport across BBB was investigated both in vitro and in vivo. In vitro study showed that, compared to the human peripheral endothelial cells, such as umbilical vein endothelial cells, lung microvascular endothelial cells, and fetal astrocytes, FC5 and FC44 bind specifically to human cerebromicrovascular endothelial cells (HCEC). Uptake study showed while the transport of 10 kDa dextran or an unrelated llama sdAb into HCEC was negligible, significant uptake of FC5 and FC44 into the HCEC was observed [7]. The polarized transcytosis of FC5 across HCEC monolayers was also reported. The study showed, in contrast to the paracellular transport marker sucrose which showed similar apical-to-basolateral (A-B) and basolateral-to-apical (B-A) transport across HCEC, the transport of FC5 across HCEC monolayers in the A-B direction was 12 times higher than that in the B-A direction (Figure 3). The transport of FC5 across HCEC was temperature dependent but not charge independent, suggesting the transport was mediated by a receptor. It is reported that the transcytosis of FC5 across human brain endothelial cells is mediated by receptor TMEM30A [76]. Additional studies indicated that following internalization, FC5 was targeted to early endosomes, bypassed late endosomes/lysosomes, and remained intact after transcytosis. FC5 endocytosis was a clathrin-mediated process which was triggered by the binding of FC5 to the *α*(2,3)-siaglycoprotein receptor [69]. Since both FC5 and FC44 are highly positively charged, the endocytosis could also be an adsorptive endocytosis process which is determined by the interactions between the positively charged FC5/FC44 and the negatively charges plasma membrane [7].

In vivo study also confirmed that FC5 and FC44 can transport across mouse BBB and accumulated in the brain following an intravenous injection [7]. In addition to brain, FC5 and FC44 accumulation in CSF after intravenous injection was demonstrated. By using a highly sensitive and specific method to quantitatively detect FC5 and FC44, the transport of FC5 and FC44 across the immortalized adult rat brain microvascular endothelial cell monolayer, and the brain delivery and distribution of FC5 and FC44 in rats, were studied [9]. In vitro study showed the A-B transport of FC5 and FC44 were much higher than those of two control heavy-chain fragments, EG2 and A20.1. In vivo studies showed that while the FC5 and FC44 had similar plasma PK as EG2 and A20.1, the CSF levels of both FC5 and FC44 were significantly higher (10–25 times) than the level of EG2 and A20.1. The CSF/plasma ratios of FC5 and FC44 showed even more pronounced differences, 20–40 times higher than those of EG2/A20.1. High CSF levels could be due to increased receptor-mediated transcytosis across either BBB, and/or choroid plexus, suggesting that they are potential novel carriers for drug delivery across the BBB and BCSFB.

sdAbs possess good properties as vectors for BBB drug delivery. They are more heat-resistant and stable towards detergents and high concentrations of urea [77]. Compared to whole antibody, sdAbs have better permeability to cellular barrier such as BBB due to low molecular weight. Lacking the Fc fragments, sdAbs do not show complement system triggered cytotoxicity and are not subject to FcRn mediated recycling. 

## 6. Neonatal Fc Receptor-Mediated Recycling and Transcytosis of Immunoglobulin G

 The hypothesis of the existence of a receptor protecting IgG catabolism was proposed by Brambell et al. in 1964 [78]. The hypothesis was later proved by the observation of a specific receptor-mediated IgG uptake and transport on the enterocyte microvillous membranes of the neonatal rat [79]. The study indicated that labeled IgGs from mouse, rat, rabbit, and human were taken up by the intestinal walls and delivered to the animal, and the transport of labeled IgG was inhibited by un-labeled IgG. In contract, little or no uptake was observed with other subclasses of human immunoglobulin, such as IgA, IgD, IgE, or IgM. The receptor was then identified as the neonatal Fc receptor (FcRn) [80, 81]. 

FcRn is a heterodimeric receptor composed of the Major Histocompatibility Complex (MHC) class 1-like heavy chain and the *β*2-microglobin light chain. It binds the Fc domain of IgG tightly at the acidic pH 6.0 and dissociates at the neutral pH 7.4. FcRn is expressed on the capillary endothelium, intestinal epithelium, and vascular endothelium [82–85]. The expression of FcRn in the vascular endothelial cells is associated with its protection of IgG against lysosomal degradation. Expression in bone marrow derived cells significantly extend the half-life of serum IgG indicating that, in addition to the vascular endothelium, bone marrow-derived phagocytic cells are a major site of IgG homeostasis [86].

Unlike most receptors which are expressed on the cell surface, FcRn primarily resides in an intracellular compartment, probably sorting endosome, with limited number on the cell surface; therefore therapeutic IgGs are required to be first taken up by cells through the fluid phase pinocytosis. Due to its rapid recycling after incomplete fusion with the plasma membrane, the amount of FcRn on plasma membrane is low [87]. A small number of bound IgG can transport to the opposite side of cell surface (i.e., transcytosis) where IgG is released. In vitro studies indicated that FcRn regulated the transport of IgG across the polarized cell monolayers that overexpressed FcRn [88]. The studies supported that FcRn can mediate the endocytosis and transcytosis of IgG in both directions. Importantly, the studies suggest that FcRn can carry bound IgG bidirectionally across endothelial barriers of blood vessels.

The protection of FcRn on IgG and albumin from degradation was demonstrated in two siblings with markedly deficiency in both IgG and albumin, and eight relatives of the siblings with moderately deficiency in IgG. The genes of the two siblings were sequenced and the results showed while the MHC class 1-like heavy chain gene sequence was normal, there was a single mutation in the gene of the *β*2-microglobin chain which caused the concentration of the soluble *β*2-microglobin chain and HLA less than 1% of the normal level. It is then concluded that it is the *β*2-microglobin mutation that resulted in the hypercatabolism and decreased the serum levels of albumin and IgG in the two siblings with familial hypercatabolic hypoproteinemia [89].

**Figure 4:**
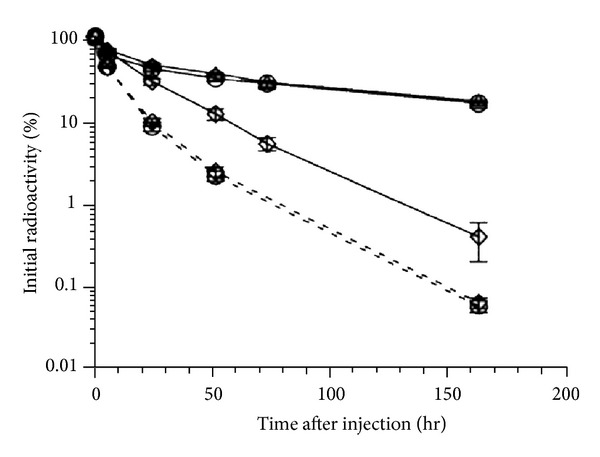
Clearance of intravenously injected ^125^I-labelled mouse IgG1 and chicken IgY antibodies in mice with and without **β**2-microglobulin. Mouse IgG1, solid line; chicken IgY, broken line; **β**2-microglobulin +/+, circles; **β**2-microglobulin +/−, triangles; **β**2-microglobulin +/−, diamonds; *n* = 5 for each group (adapted from Figure 1, [90]).

The importance of the *β*2-microglobin subunit of FcRn in maintaining the exposure of IgG was also demonstrated in animal studies. It was found that the clearance of all subclasses of mouse ^125^I-labelled IgG, with the possible exception of IgG2b, was strikingly more rapid in the *β*2-microglobulin-deficient mice than that in the heterozygous or the wild-type mice. To confirm that the faster clearance of IgG was due to the deficient FcRn, the clearance of a chicken IgY, which does not bind FcRn, was also examined. As shown in Figure 4, the clearance of the ^125^I-labelled IgY was similar in the *β*2-microglobulin-deficient and the wild-type mice [90]. The essential role of the *β*2-microglobin subunit in maintaining functional FcRn was also confirmed in another study which showed the clearance rate of IgG increased by 10 times in *β*2-microglobulin deficient mice in comparison to the wild-type mice [91]. 

 Amino acids of FcRn and at the CH2-CH3 domain of IgG that are crucial for the interactions between FcRn and IgG have been identified [92, 93]. Substitution of these amino acids disrupted the affinity between FcRn and IgG [94]. It is noted that the interactions between FcRn and IgG are also determined by the Fab domain of IgG. It has been reported that FcRn bound with remarkable differences to IgGs with the wild-type human Fc domain but different Fab domains. The Fab domain affected both the binding at the acidic pH and the dissociation at the neutral pH. Pharmacokinetic study in human FcRn mice, nonhuman primates, and humans showed, however, that there was an apparent correlation between the PK of the IgGs with the dissociation at the neutral pH, but not with the binding at the acidic pH [95]. 

**Figure 5:**
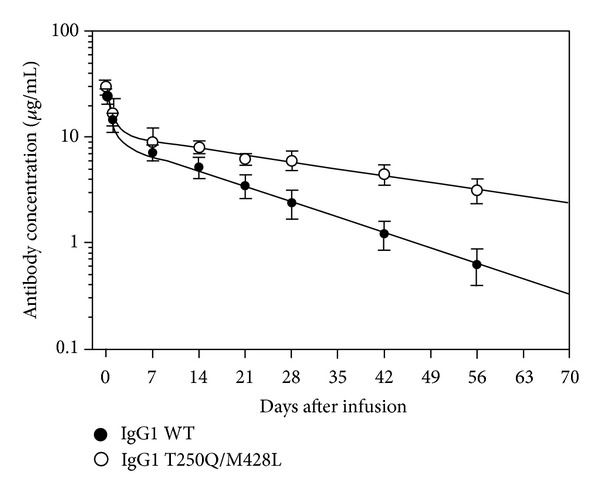
PK profile of OST577-IgG1 WT and mutant Abs following intravenous injection to rhesus monkeys. The concentration of OST577-IgG1 Abs in rhesus serum was measured in a validated ELISA using the mouse anti-OST577 anti-Id mAb for capture and the HRP-conjugated goat anti-human *λ* L chain Ab for detection (adapted and modified from Figure 2, [99]).

 Because of the protective effects of FcRn for IgG, FcRn is becoming a promising target for enhancing protective humoral immunity, treating autoimmune disease, and improving drug efficacy [96, 97]. Modulating the interaction between Fc and FcRn through protein engineering has been applied to improve the PK of the therapeutic antibodies. Various studies have shown that the prolonged half-life and exposures of the therapeutic antibodies can be achieved by increasing the pH dependent binding affinity between Fc and FcRn. The humanized antirespiratory syncytial virus (RSV) monoclonal antibody (MEDI-524) was engineered with a triple mutation of M252Y/S254T/T256E (YTE). The mutation resulted in 10 times increase in the binding affinity to both cynomolgus monkey and human FcRn at the acidic pH 6.0 but did not affect the dissociation at the neutral pH 7.4. Compared with the wild-type MEDI-524, MEDI-524-YTE showed approximately 4 times increase in serum half-life when evaluated in cynomolgus monkeys [98]. The fact that increased binding affinity at the acidic pH 6.0 can be translated to prolonged exposure is also observed with a double mutant (T250Q/M428L) on a human IgG1 antibody. The double mutant showed approximately 3 times increased binding affinity to both cynomolgus monkey and human FcRn at pH 6.0 without affecting the dissociation at the neutral pH. Pharmacokinetic study in cynomolgus monkeys showed that the serum half-life of the double mutant increased by about 2.5 times (Figure 5) [99]. These two studies suggested that, in order to prolong the half-life of the therapeutic antibodies, protein engineering on the therapeutic antibodies should only aim to increase the binding affinity to FcRn at the acidic pH 6.0, and leave the dissociation at the neutral pH unchanged. This was further demonstrated in the pharmacokinetic study of two human IgG1 Fc variants, N434A and N434W. N434A and N434W mutations resulted in 4 and 80 times increases in the binding affinity to both human and nonhuman primate FcRn, respectively. However when evaluated in cynomolgus monkeys, only the N434A mutant showed 2 times improvement in the half-life, while the half-life of the N434W mutant was similar to that of the wild-type human IgG. Further analyses indicated that the N434W mutation increased the binding affinity to FcRn not only at pH 6, but also at pH 7.4. The study emphasizes that modest increases of the affinity to FcRn at acid pH 6 but not neutral pH 7.4 can result in improved PK [14]. The lack of improved PK or even reduced PK for variants with increased affinity at the neutral pH 7.4 may be due to the fact that the increased binding at pH 7.4 hinders the release of the variants from FcRn into circulation, therefore canceling out the benefit of increasing affinity at pH 6.

Because of the high expression levels in a variety of tissues including vascular endothelial cells, bone marrow, skin, and muscle [86, 100], the function of FcRn could not be readily saturable. In fact, saturation of FcRn is only observed when overload of exogenous of IgG or serum albumin. The impact of intravenous immunoglobulin (IVIG) therapy on the PK of an anti-platelet antibody, 7E3, was evaluated in FcRn deficient mice. In the study, mice were dosed 1 g/kg IVIG followed by 8 mg/kg 7E3. IVIG administration increased the clearance of 7E3 by about 3 times in the wild-type mice, while showing no effect of the clearance in FcRn deficient mice. The results indicate that the increased clearance of 7E3 in the wild-type mice was due to the saturation of FcRn by high dose IVIG administration [101]. 

 FcRn has been shown to transport IgG across cellular barriers, including those in brain, intestine, and placenta [96, 102, 103]. The Fc domain of IgG has been utilized as a vehicle for efficiently delivery of therapeutic proteins with prolonged retention and biological activity. The therapeutic proteins are fused to the Fc domain, allowing them to bind to FcRn. Various recombinant fusion proteins have been engineered by conjugating the Fc domain to proteins such as growth factors, cytokines, and enzymes to achieve prolonged half-live and therapeutic effects [104–106]. Due to the short half-live (10–12 hr), factor VIII has to be administrated 3 times per week to reach full prophylaxis [107]. To reduce the dose frequency, a recombinant fusion protein was engineered by fusing the Fc domain of IgG1 to factor VIII. PK studies showed that both half-life and the efficacy duration of the factor VIII-Fc fusion protein increased by approximately 2 times in comparison to the unmodified factor VIII when evaluated in hemophilia A dogs and hemophilia A mice expressing either the endogenous murine FcRn or transgenic human FcRn. In contrast, the increased half-life and efficacy duration were not observed in FcRn knocked out mice, indicating that the enhanced exposure and efficacy duration were mediated by FcRn [13]. 

In vitro study using immortalized rat brain endothelial cells suggested that the human Fc fragment transports faster in brain-to-blood direction than in blood-to-brain direction. The study showed that while FcRn mediated the transport of IgG across peripheral vascular cells in both directions, FcRn only mediated transport across BBB in brain to blood direction. The clearance of human Fc, BSA and 10 kDa Dextran was compared in vivo following intracerebral injection. The results indicated that the residence half-lives of human Fc and BSA were 2.2 and 1.5 h, respectively, shorter than the 4.1 h half-life of the 10 kDa dextran [15]. The expression of FcRn in brain has been confirmed. Confocal microscopy confirmed that FcRn is expressed throughout the rat cerebral microvasculature, including the brain capillary bed, and pre-capillary arterioles. Colocalization with the Glut1 glucose transporter indicates that the brain microvascular FcRn is expressed in the capillary endothelium, likely localized to either the endothelial abluminal and/or luminal membrane [83].

Since FcRn is expressed on the brain capillary endothelium, it has been proposed that the efflux of IgG from brain to blood is mediated by FcRn. A study in rats showed intracerebral injected IgG was rapidly efflux from brain to blood with half-life of 48 min. The efflux was inhibited by IgG, but not rat albumin. Furthermore, only the Fc fragments but not the Fab fragments inhibited the efflux [16]. This study suggests that BBB FcRn mediates the efflux of IgG from brain to blood. 

 The role of FcRn in regulating the efflux of IgG from brain is also suggested by the study to investigate the mechanism of A*β* immunotherapy in the clearance of A*β* amyloid peptide. In the study, the effects of peripherally and centrally administered A*β*-specific IgG on the influx of circulating A*β* amyloid peptide from blood to brain, and the efflux of brain-derived A*β* amyloid peptide from brain to blood, were studied using both the APPsw(+/−) mice, a model that develops Alzheimer's disease-like amyloid pathology, and the wild-type mice. The study showed that anti-A*β* IgG blocked the influx of circulating A*β* amyloid peptide from blood to brain in APPsw(+/−) mice. In young mice, the complexes of A*β* amyloid peptide and anti-A*β* IgG were cleared from brain to blood by both FcRn and LRP mediated transcytosis across the BBB; while in older mice, FcRn played a more important role in the efflux A*β* of amyloid peptide from brain to blood. The anti-A*β* IgG assisted efflux of A*β* amyloid peptide from brain to blood in the wild-type mice was inhibited when the FcRn gene was knocked out. The study indicated that FcRn at the BBB plays a role in regulating IgG-assisted A*β* amyloid peptide removal from the aging brain [103].

 However conflicting studies suggested that the brain disposition of IgG is not regulated by FcRn. In the study using the *β*2-microglobulin knock-out mice, ^125^I-labeled 7E3, a monoclonal IgG1 antibody, was injected intravenously to FcRn deficient mice and control mice. The blood and brain exposures were determined. As anticipated, the plasma clearance of 7E3 was increased by about 10 times and the plasma exposures decreased by 4-5 times in FcRn deficient mice when compared to the control mice. However the brain exposure of 7E3 was also reduced to a similar extent; as a result, the brain to plasma ratios of 7E3 were not significantly different between the FcRn deficient mice and the control mice [18]. Since *β*2-microglobulin is a subunit of multiple proteins in addition to FcRn, it might be inconclusive if the results obtained solely resulted from FcRn deficiency, therefore the role of FcRn in regulating brain IgG disposition was further investigated. In this study, the distribution of 8C2, a murine monoclonal IgG1 antibody, was evaluated in the FcRn *α*-chain knockout mice, FcgammaRIIb knockout mice, FcgammaRI/RIII knockout mice, and C57BL/6 control mice. Following intravenous injection to the mice, the blood and brain exposures of 8C2 were determined. Compared to that from the control mice the plasma and brain exposures from FcgammaRIIb knockout mice, and FcgammaRI/RIII knockout mice were not significantly different, and the plasma and brain exposures from FcRn *α*-chain knockout mice decreased by 3-4 times as anticipated. However, similar to what was observed in the previous study [18], the brain to blood exposure ratio was not significantly different among the knockout and control mice [17]. Together, both studies indicated the BBB FcRn does not regulate the efflux of IgG across the BBB.

## 7. Conclusion

Receptor-mediated endocytosis and transcytosis are the fundamental processes which proteins are taken up and transported across the endothelial and epithelial cells. With the identification of new ligands and antibodies against the receptors expressed on the brain capillary endothelial cells, receptor-mediated brain delivery of DNAs, peptides, and proteins has been achieved by using the Angiopep-2-conjugated systems and the molecular Trojan horses. Since receptor-mediated endocytosis is generally a saturable process, receptor-mediated brain delivery could interfere with the intended function of the receptors, especially following chronic high dose administration. 

The function of FcRn in regulating IgG recycling and protecting IgG against lysosomal degradation has been well characterized. The improved PK of therapeutic IgGs has been achieved by increasing the pH dependent binding affinity to FcRn. FcRn is also expressed on BBB. Elucidating the BBB FcRn function will provide insight in designing therapeutic IgG antibodies and molecular Trojan horses to achieve rapid brain delivery, prolonged brain exposures, and rapid removal of the targets in some cases, such as A*β* amyloid plaque, from brain. FC5 and FC44 are promising vectors for brain delivery. Whether lacking the Fc domain, therefore the interaction with FcRn, is necessarily a good feature is debatable as it depends on the pharmacology of the therapeutic proteins as well as the function of BBB FcRn in determining the disposition of molecules containing the Fc domain in the brain.
